# The Molecular Regulation in the Pathophysiology in Ovarian Aging

**DOI:** 10.14336/AD.2020.1113

**Published:** 2021-06-01

**Authors:** Chia-Jung Li, Li-Te Lin, Hsiao-Wen Tsai, Chyi-Uei Chern, Zhi-Hong Wen, Peng-Hui Wang, Kuan-Hao Tsui

**Affiliations:** ^1^Department of Obstetrics and Gynaecology, Kaohsiung Veterans General Hospital, Kaohsiung, Taiwan.; ^2^Institute of BioPharmaceutical sciences, National Sun Yat-sen University, Kaohsiung, Taiwan.; ^3^Department of Obstetrics and Gynaecology, National Yang-Ming University School of Medicine, Taipei, Taiwan.; ^4^Department of Marine Biotechnology and Resources, National Sun Yat-sen University, Kaohsiung, Taiwan.; ^5^Department of Obstetrics and Gynecology, Taipei Veterans General Hospital, Taipei, Taiwan.; ^6^Department of Medical Research, China Medical University Hospital, Taichung, Taiwan.; ^7^Female Cancer Foundation, Taipei, Taiwan.; ^8^Department of Pharmacy and Master Program, College of Pharmacy and Health Care, Tajen University, Pingtung County, Taiwan.

**Keywords:** ovary, aging, pathophysiology

## Abstract

The female reproductive system is of great significance to women’s health. Aging of the female reproductive system occurs approximately 10 years prior to the natural age-associated functional decline of other organ systems. With an increase in life expectancy worldwide, reproductive aging has gradually become a key health issue among women. Therefore, an adequate understanding of the causes and molecular mechanisms of ovarian aging is essential towards the inhibition of age-related diseases and the promotion of health and longevity in women. In general, women begin to experience a decline in ovarian function around the age of 35 years, which is mainly manifested as a decrease in the number of ovarian follicles and the quality of oocytes. Studies have revealed the occurrence of mitochondrial dysfunction, reduced DNA repair, epigenetic changes, and metabolic alterations in the cells within the ovaries as age increases. In the present work, we reviewed the possible factors of aging-induced ovarian insufficiency based on its clinical diagnosis and performed an in-depth investigation of the relevant molecular mechanisms and potential targets to provide novel approaches for the effective improvement of ovarian function in older women.

## 1.Clinical diagnosis of ovarian insufficiency

Decreases in oocyte quantity and quality induced by ovarian insufficiency, which is generally manifested as the depletion of the primordial follicle pool, are the main influencing factors of pregnancy in women. Most women experience decreased ovarian function around the age of 50 years [1]. Ovarian insufficiency increases the risk of developing a series of complications such as osteoporosis, cardiovascular diseases, recurrent depression, and cognitive impairment, thereby leading to a decreased quality of life [2, 3]. At present, the key diagnostic indicators of ovarian insufficiency include age, follicle-stimulating hormone (FSH) levels, anti-Mullerian hormone (AMH) levels, inhibin B levels, antral follicle count (AFC), ovarian stromal blood flow, and baseline ovarian volume [4]. Despite the widespread use of AMH and AFC levels in the diagnosis of ovarian insufficiency, none of the above-mentioned indicators are an independent predictor of ovarian function [5]. The age-appropriate decrease in functional ovarian reserve within a given range is known as normal ovarian aging. Studies have shown that approximately 10% of women develop occult premature ovarian insufficiency, which is an age-inappropriate decrease in functional ovarian reserve without the manifestation of significant clinical symptoms [5, 6]. Approximately 1% of women develop premature ovarian insufficiency (POI), which is characterized by the premature depletion of ovarian follicles or amenorrhea before the age of 40 years [7]. The European Society of Human Reproduction and Embryology recommends the following criteria for the diagnosis of POI: (i) amenorrhea for at least 4 months and (ii) an elevated FSH level >25 U/L. For premature ovarian failure, which is characterized by more severe clinical manifestations, the diagnostic criteria include an elevated FSH level >40 U/L accompanied by secondary amenorrhea for at least 4 months [8].

## 2.Etiological factors of aging-induced ovarian insufficiency

### 2.1 Mitochondria and mitochondrial DNA

Mitochondria play the role of energy generators in cells. They are tubular in shape and consist of an outer membrane, inner membrane, cristae, and matrix. Human mitochondrial genetic material (mtDNA) is approximately 16.6 kb long, double-stranded, circular and lacks histones. It contains 37 genes encoding 2 mitochondrial rRNAs, 22 tRNAs, and 13 oxidative phosphorylase complex protein subunits. Mitochondria are the most abundant organelles in the egg cytoplasm. They not only provide energy for oocyte maturation, fertilization, and embryo development, but also regulate calcium ion homeostasis, apoptosis, autophagy, and other life activities. Abnormal numbers and functions affect the outcome of fertilization and embryo development [9, 10].

#### 2.1.1. mtDNA copy number in oocytes

The quantity of mitochondria in oocytes affects reproduction. In somatic cells, each mitochondrion contains 1-15 copies of mtDNA [11], while in oocytes, each mitochondrion contains only one copy of mtDNA [12]. Oocytes dormant in primordial follicles contain less than 10,000 mitochondria [13], and mid-ovulatory MII eggs can have up to 1.5 million mitochondria [14]. Oocytes are the largest cells in the female body and mature oocytes contain more mtDNA than other somatic cells [15]. The number of mitochondria increases during meiosis in oocytes, and the mid-ovulation MII egg reaches its peak, with 100,000 mitochondria and 50,000 to 550,000 copies of mtDNA. Mature oocytes have the highest mitochondrial content in human cells, which may be due to the huge energy demand in oocyte fertilization and early embryo development [15].

#### 2.1.2. mtDNA and oocyte quality

Decreased mtDNA content may cause failure of oocyte *in vitro* fertilization [16]. The content of mtDNA in unfertilized oocytes is significantly reduced compared with normal fertilized oocytes. The lack of spindle formation during meiosis may result in low mtDNA content and ultimately poor embryo quality [17]. In the MII phase, the mtDNA content is negatively correlated with the age of women, and the increase in women’s age leads to a decrease in the number of mtDNA in oocytes. Decrease and mutation rate increase, which in turn leads to a decrease in its developmental potential [18]. The mutation rate of mtDNA in granule cells (CCs) of oocytes in elderly patients is higher, and the evaluation of mtDNA content in granule cells has the ability to predict embryo quality [19, 20]. Studies have found that there is a high rate of mtDNA deletions in IVF female xanthogenic granular cells [21]. Therefore, it is proposed that age-related infertility is primarily related to reduced oocyte development potential.

#### 2.1.3.Mitochondrial dysfunction

Mitochondria are the key factor affecting the quality of oocytes and are essential for providing sufficient ATP, but these may be directly affected during ovarian aging [22, 23]. Aging increases mitochondrial DNA (mtDNA) instability, which causes the accumulation of mtDNA mutations in cells within the ovaries, especially in oocytes. Mitochondria play an essential role in ovarian follicle development and early embryonic development, and a decline in ovarian function also severely affects mitochondrial biogenesis and function in oocytes and peripheral granulosa cells [24]. Therefore, mitochondrial dysfunction in oocytes, which possess the highest mtDNA copy number among all cells, accelerates ovarian insufficiency, resulting in pregnancy failure. Morphological and functional studies have found that aging affects mitochondrial function in cells, especially oocytes, leading to mitochondrial swelling, vacuolization, and fragmentation [25, 26]. Reactive oxygen species (ROS) are the leading cause of mtDNA mutations [27]. The mitochondrial free radical theory of aging proposes that aging causes the accumulation of large amounts of oxygen free radicals and ROS, which leads to mtDNA mutations and consequently affects the operation of the electron transport chain (ETC). Additionally, mtDNA mutations further aggravate the accumulation of ROS and mtDNA mutations, thereby causing a cycle that can ultimately result in reduced ATP yield, cell cycle arrest, or even apoptosis. Studies have shown that in addition to ROS, various disorders of mitochondrial dynamics such as mitochondrial fusion, ETC inactivation, changes in mitochondrial metabolism, and imbalance of calcium homeostasis [28-30] are associated with oocyte aging. In a study by Latorre-Pellicer et al. a multi-omics approach was used to analyze changes in mtDNA during the aging process in mice, and the results revealed that single-locus changes in mtDNA could influence mitochondrial proteostasis, accelerate ROS generation, and result in telomere shortening [30]. Mitofusin 2 (MFN2), a GTPase embedded in the outer mitochondrial membrane, is an essential protein for mitochondrial fusion. In a previous study, severe developmental delays and embryonic deaths caused by placental defects were observed in *Mfn2* knockout mice, and infertility occurred in female mice after oocyte-specific knockout of *Mfn2* [31]. The mitochondria fission-regulating GTPase dynamin-related protein 1 (Drp1) plays a key role in maintaining normal function in reproductive cells. A study by Udagawa et al. showed that oocyte-specific knockout of *Drp1* led to defective follicular maturation and ovulation in mice [32]. Other studies have shown that proteases involved in mitochondrial quality control, including CLPP, AFG3L2, PHB, OMA1, LONP1, and PARL, also play crucial roles in ovarian cells, as deficiencies in these proteases lead to the onset of mitochondrial-related diseases and accelerate oocyte aging [33-35]. Multidrug resistance transporter-1 (MDR-1) is known for its pathological role in tumor escape during chemotherapy. Mutations in MDR-1 can destroy the homeostasis of mitochondria in oocytes and ovaries. The function of MDR-1 reveals the key intersection of metabolite regulation, oxidative stress, and mitochondrial dysfunction, which directly affects human infertility, premature reproductive aging, and gonadal protection due to oxidative stress [36].

#### 2.1.4. mtDNA and fertility

Decreased mtDNA content may cause in vitro fertilization failure of oocytes in IVF [16]. Compared with normal fertilized oocytes, the content of mtDNA in unfertilized oocytes was significantly reduced. The lack of spindle formation during meiosis may result in low mtDNA content and ultimately poor embryo quality [17]. In the MII phase, the mtDNA content is negatively correlated with the age of women, and the increase in women’s age will lead to a decrease in the number of mtDNA in oocytes. Decrease and mutation rate increase, which in turn leads to a decrease in its developmental potential [18]. The mutation rate of mtDNA in granule cells (CCs) of oocytes in elderly patients is higher, and the evaluation of mtDNA content in granule cells has the ability to predict embryo quality [19, 20]. Studies have found that there is a high rate of mtDNA deletions in IVF female xanthogenic granular cells [21]. Therefore, it is proposed that age-related infertility is primarily related to the weakened oocyte development potential.

Previously published studies have shown methods to establish clinically relevant mtDNA parameters to predict embryo viability. For example, Fragouli et al. [37]identified a critical point above which pregnancy does not occur, while Diez-Juan et al. [38] pointed out that a scale is proposed where the potential for implantation is reduced in relation to increased mtDNA . Fragouli’s initial publication indicated that the number of copies of mtDNA was increased in relation to women’s age, while Diez-Juan [38] did not find that age had any effect on the amount of mtDNA in blastocysts. In a subsequent study, the report pointed out that mtDNA content increased with age [39], and another study pointed out that patients over 40 years old have significantly higher mtDNA content [40], while patients 21-22 years old The content of mtDNA is lower [41], whereas Victor [42] found no correlation. In general, the correlation between female age and mtDNA copy number in euploid embryos is an inconsistent finding.

These results indicate that different research methods will affect the predictive power of mtDNA. However, recent research by Richard showed that the relative mitochondral DNA copy number (RmtDCN) in human euploid blastocysts cannot predict reproductive outcomes. This investigation emphasizes that RmtDCN may not be a useful parameter, especially in clinical centers with stable laboratory conditions, which may prevent excessive increases in mtDNA, as previously recommended by Ravichandran [41]. In addition, even in animal models with clear mitochondrial function and dysfunction, the mtDNA copy number determined by quantitative PCR shows significantly higher variability and limited ability to identify mitochondrial dysfunction [43].

### 2.2. Genetic changes

Declining ovarian function is associated with decreased oocyte quality. From birth onwards, the ovaries of a woman are constantly affected by factors such as hormones, metabolism, and immunity during the entire female growth and development process, thereby leading to the occurrence of DNA damage in oocytes and somatic cells within the ovaries. Previous research has revealed that as age increases, the number of DNA double-strand breaks (DSBs) in oocyte nuclei increases significantly and the expression of DNA repair genes in the ovaries is reduced, which leads to the continuous accumulation of DSBs [44]. During aging-induced meiotic processes, deficiencies in chromosomal cohesin affect chromosome segregation in oocytes, thereby causing an increase in the proportion of aneuploid oocytes and influencing oocyte function [45]. Additionally, during sister chromatid separation, the centromere is subjected to traction forces in two opposite directions from spindle fibers that may lead to erroneous separation, which may be another significant cause of oocyte aneuploidy [46]. As the spindle assembly checkpoint (SAC) prevents chromatid separation until the proper attachment of sister chromatids on the mitotic spindle is accomplished, defects in the SAC can also cause a significant increase in aneuploidy rate [47, 48]. One study observed that the aneuploidy rate in oocytes of aged mice was 31.6%, whereas the rate in young mice was 4.9% [49]. Telomere shortening commonly exists in aged oocytes mainly due to an aging-induced increase in the ROS content of the cells [24, 50] and has been shown to contribute to the DNA damage response [51]. The quality of granulosa cells in the periphery of oocytes also affects oocyte quality.

**Table 1:** Alterations in gene expression and activity of pathways involved in the aged oocyte.

Gene name	Gene function	Ref.
CREB1	primordial follicle activation & Regulator in Senescent Granulosa Cells	[24, 127]
MAPK3/1	primordial follicle activation	[128]
TFAM	human oocytes maturation	[129, 130]
NRF1	early bovine embryogenesis	[129-131]
PGC-1α	Follicular development	[131]
Peroxiredoxin 2 (Prdx2)	cumulus expansion and oocyte maturation	[132]
type I interferons (IFNs)	induction of cumulus expansion	[133]
Has2	cumulus-oocyte complexes	[134, 135]
PTX3	follicular growth	[136]
TrkA	cumulus-oocyte complexes	[137]
PI3K	in vitro maturation and developmental	[138]
COX-2	in vitro maturation	[139]
BMP-15	oocyte development and functions	[140-143]
GDF9	cumulus cell expansion and oocyte competency	[140, 142-144]
FGF10	cumulus expansion, glucose uptake	[141]
PGR	cumulus cell expansion	[135]
Ganglioside GD1a	oocyte maturation and preimplantation development	[145]
Ganglioside GM3	Reduced cumulus cell apoptosis	[146]
Ganglioside GT1b	Oocyte Maturation & Cumulus Cell Expansion	[147]
(natriuretic peptide precursor C Nppc)	Regulation of Oocyte Meiotic	[148]
SIRT1	oocyte aging	[149]
Protein kinase D (PKD)	Maintain spindle formation and actin assembly	[150]
Ubiquinol-10	delay oocyte aging	[151]
MFN2	oocyte and follicle development	[31]
NPR2	maintains oocyte meiotic	[152, 153]

With increasing age, cumulus and granulosa cells also experience an increase in DNA damage and telomere shortening [24, 52-54]. Multi-omics studies have shown that aging may cause significant changes in genes within oocytes involved in cell-cycle signal transduction and changes in the expression of proteins related to SAC, DNA stability, chromatid separation, cell division, microtubules, and regulatory RNAs [55-57]. In a microarray study on the primordial follicles of aged and young rats, the differences in the expression of molecules related to nucleotide binding, RNA binding, structural constituent of ribosome, transcription factor binding activity, cell cycle, homologous recombination, meiosis, DNA replication, and the MAPK signaling pathway between the aged and young animals were found to be significant at the transcription level [58]. Similar variation trends were obtained in studies on other species such as wild cattle, mice, and the domestic goat. In particular, oocytes derived from aged cows exhibited high expression of molecules related to the eukaryotic initiation factor 2 (eIF2) signaling pathway [58-61]. Research has shown that changes in many genes affect oocyte quality and accelerate ovarian aging. For instance, mutations in the breast cancer 1 gene (*BRCA1*), which participates in meiotic spindle assembly, accelerate the decline in ovarian function in women [61, 62]. In another study, it was found that the expression level of the structural maintenance of chromosomes 5/6 (SMC5/6) complex in mice decreased with increasing age, and the age-dependent depletion of the SMC5/6 complex led to a significant decrease in the oocyte aneuploidy rate [63]. Table 1 shows the genes in oocytes which undergo significant changes with an increase in age.

### 2.3. Changes in cytoskeleton and microtubules

The chromosome segregation error during female meiosis is the main cause of pregnancy loss and human infertility. The separation of chromosomes is driven by the interaction between spindle microtubules and kinetochores. Previous studies have found that the centromere chromatin disappeared with the increase of maternal age. Kintochores built on damaged centromeres frequently lost their integrity and split into multiple lobes. The fragmented kinetochores were abnormally attached to spindle microtubules, which indicates that the fragmentation of kinetochores may be related to the maternal age effect of mammalian eggs [64]. The oocytes of naturally aging mice exhibited significantly altered spindle microtubule dynamics, resulting in a transient multipolar spindle, which makes the oocytes prone to form kinetochore-microtubule attachment defects and complete sister staining lists Disaggregation of body pairs. The current hypothesis is that although the loss of cohesion can explain the premature sister separation, the classic non-separation phenomenon can be explained by changes in microtubule dynamics, leading to abnormal spindle assembly [65]. The latest research indicates that a non-synonymous variant of centrosomal proteins (CEP120) disrupted female meiosis in mice. The study used the mouse oocyte system for functional verification. Because mouse and human oocytes have different spindle construction methods. Therefore, the defects observed when ectopic expression of Cep120 variants may change the meiosis of mouse oocytes differently from the meiosis of human oocytes. It was found that patients with a high proportion of aneuploidy blastocysts carry a higher mutation burden in the functional genes of the cytoskeleton and microtubule pathway [66].

With the increase of reproductive age, not only the non-separation of meiosis that occurs is attributed to cytoplasmic defects in the microtubule cytoskeleton [65], but also the changes in chromosome-related proteins (such as: cohesins and those at the kinetochore) can lead to chromosomes Separation error [67]. In addition, the senescence of oocytes has been shown to be related to transzonal projections (TZPs). The transzonal projections (TZPs) are the filopodia in the cytoskeleton. The number of TZPs will increase significantly and accompanied by the growth of oocytes, thereby enhancing the communication between cumulus oocyte complex (COCs). The close packing of granulosa cells may also restrict the growth of TZP on the surface of the oocyte, and TZP-like filaments extending toward the oocyte. They are likely to establish stable interactions after TZP reaches the surface of the oocyte, as shown by the presence of *zonula adherens* [68] and desmosome-like junctions [69] at the contact points. The growth differentiation factor 9 (GDF9) produced by oocytes promotes the formation of new TZPs and further enhances the communication and exchange between granulosa cells and oocytes [70]. By providing evidence that the defect in germline-soma communication may be the basis of age-related oocyte quality decline, this is of great significance for understanding female infertility. Although it is generally believed that the decline in oocyte quality is germ cells, the elderly produced in granulosa cells are also defective. This result identified a specific defect in the germline-soma interaction in elderly women and linked it to the decreased expression of related genes in granulosa cells. Although this decrease may be due to impaired oocyte signaling, it may indicate that the primary foci of poor oocyte quality originated in the somatic compartment of the follicle [71, 72].

### 2.4. Molecular regulation of epigenetics

The establishment of proper epigenetic modifications during oocytogenesis and early embryonic development is a crucial aspect of reproduction. Studies have shown that improper epigenetic modifications occur in DNA in reproductive cells during the aging process, including abnormal DNA methylation, histone modifications, and non-coding RNA-regulated modifications. These epigenetic modifications play unique roles in the regulation of oocyte aging [73].

#### 2.4.1 DNA methylation

DNA methylation in oocytes plays an essential role in the development of the reproductive system. In a study investigating the mRNA expression profiles of oocytes from young and aged mice, differences at the 5% significance level were found in the transcription of genes encoding proteins involved in epigenetic modification, chromatin remodeling, and DNA methylation, including DNA methyltransferase 1 (DNMT1), DNMT3A, DNMT3B, DNMT3L, and DNA methyltransferase 1-associated protein 1 (DAMP1) [74]. Similar transcript changes were observed when gene expression microarray analysis was performed on another mouse strain [58]. Aging promotes the high expression of DNMTs in oocytes, which in turn catalyze DNA methylation. Previous research has indicated that stillbirth and fetal malformation rates are significantly higher in aged mice than those in young mice, which is closely associated with abnormal DNA methylation in oocytes [75]. DNMTs also exert regulatory effects on CpG sites in the genome. Studies have shown that three members of the p53 family (p53/p63/p73) may potentially play a role in oocyte aging. In particular, the p73 gene encodes two major protein isoforms, namely TAp73 and ΔNp73. In humans, TAp73 expression, which is regulated by DNA methylation patterns, was found to be significantly lower in the oocytes of women older than 38 years of age compared with the oocytes of women younger than 36 years of age [9]. However, there is a current lack of direct evidence of an increased influence on DNA methylation in human oocytes. DNA methylation can also occur at sites other than CpG sequences, which is known as non-CpG methylation or asymmetric methylation. A recent study that adopted the single-cell whole-genome bisulfite sequencing technique for the investigation of DNA methylation patterns in human oocytes revealed the presence of high levels of non-CpG methylation in human oocyte DNA. With oocyte maturation, non-CpG methylation in the genome gradually accumulates, but the biological roles that non-CpG methylation may play in these specific cell types remain to be investigated. Future research on single-cell transcriptomic changes associated with human oocyte maturation may help to elucidate the physiological roles played by non-CpG methylation during human oocyte maturation [76].

#### 2.4.2 Histone modifications are involved in regulation of oocyte aging

DNA methylation, while being the most common epigenetic mark, is also linked to other epigenetic marks [77]. Histone modifications, including methylation, acetylation, ubiquitination, and other modifications, are also important epigenetic modifications. In particular, histone acetylation regulates cell functions such as chromosome condensation, DNA break repair, and transcription [78-80]. During the maturation of mammalian oocytes, acetylation occurs on histones H3 and H4. Previous research has revealed the occurrence of significant changes in gene expression and histone acetylation in the oocytes of aged animals compared with younger oocytes [81]. The methylation of histone H3 at lysine 4 (H3K4) is usually associated with gene activation and aging [82, 83], with H3K4 methylation levels being higher in the MII-stage oocytes of younger animals than those in aged oocytes. In another study, the expression of the histone methylation-related factors CBX1 and SIRT1 exhibited opposite trends in the germinal vesicle (GV)-stage oocytes of aged animals, with CBX1 expression demonstrating a significant increase and SIRT1 expression exhibiting a remarkable decrease [84].

Besides methylation patterns, histone acetylation in human oocytes also exhibits changes with increasing age. One study found that human GV-stage oocytes demonstrated intense staining of the chromatin for the acetylation of various histone 4 lysines (h4k5, h4k8, h4k12, and h4k16), whereas the MI-stage and MII-stage oocytes showed different deacetylation levels in the four histone 4 lysines [85]. The results of the study also showed that the amount of defective histone acetylation during human oocyte meiosis increased with maternal age. In particular, advanced maternal age was associated with increased H4K12 acetylation in oocytes and negatively influenced H4K12 deacetylation in MII-stage oocytes [85].

**Figure 1.**
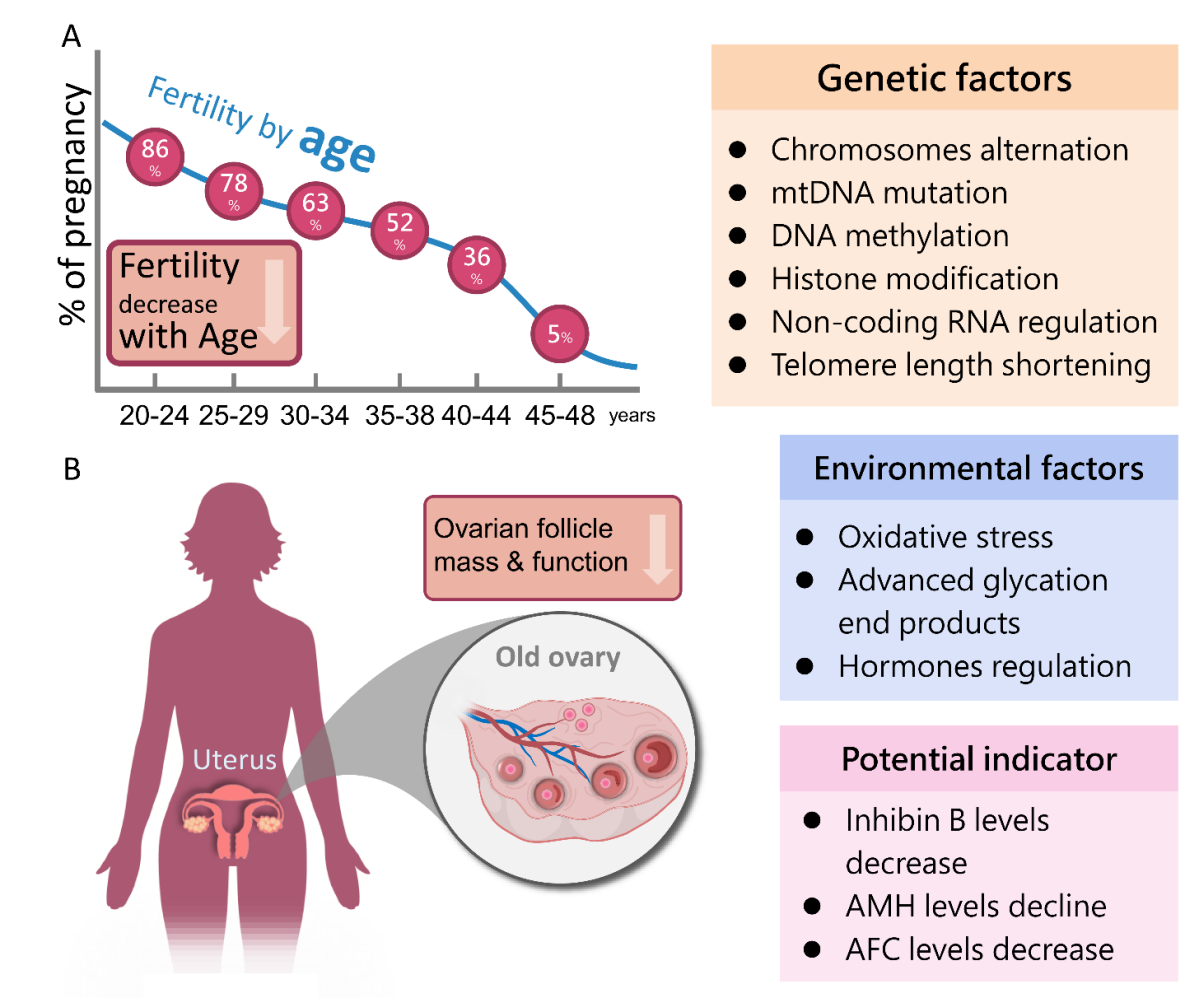
The prevalence and related factors of ovarian dysfunction and aging. (A) Trends in age and female fertility. (B) The key factors affecting the decline of ovarian function include: functional ovarian aging, environment, and heredity. Chromosomal abnormalities, mitochondrial DNA mutations, post-gene modification and changes in telomere length are all important factors for gene regulation of ovarian teachers. The microenvironmental factors include: oxidative stress, advanced glycation end products, hormones regulation. The current clinical potential evaluation indicators were: Inhibin B, AMH and AFC levels.

Histone ubiquitination, which is also affected by the aging of human oocytes, decreases with increasing age. A previous study comparing the mRNA expression profiles of oocytes from women aged 37-39 years and women aged <36 years found that biological processes such as cell cycle and protein ubiquitination were linked to oocyte aging [55]. In women aged 37-39 years, genes for the ubiquitin-specific peptidases USP2, USP34, and USP42 were upregulated [55], indicating that human oocyte aging might influence ubiquitination. The occurrence of hypoxia in granulosa cells of aged women results in the generation of ROS and oxidative stress, which induces autophagy in granulosa cells and oocytes. Therefore, hypoxia is the leading cause of ovarian aging and decreased oocyte quality [33]. Hypoxia has also been shown to decrease H3K4 methylation, increase H3K9 methylation, and simultaneously decrease H3K9 acetylation in the *BRCA1* promoter of the DNA damage response signaling pathway [86]. These results provide possible hypotheses for the epigenetic changes associated with oocyte aging (Fig. 1).

#### 2.4.3 microRNA regulates aging oocytes

Besides DNA methylation and histone modifications, non-coding RNA-directed epigenetic modifications also constitute a key epigenetic mechanism. MicroRNAs (miRNAs), which are small non-coding RNA molecules, bind specifically to target mRNAs through sequence-specific interactions, thereby inhibiting the translation or degradation of the target mRNAs and regulating protein translation at the post-transcriptional level [87]. MiRNAs, which are abundant in human MII-stage oocytes and peripheral cumulus cells, may serve a role in regulation of the interactions between oocytes and cumulus cells [88]. In a study on miRNAs expressed in human MII-stage oocytes, it was found that 12 miRNAs were involved in the regulation of the oocyte aging process. The results showed that three miRNAs (let-7b-5p, miR-19a-3p, and miR-519d-3p) were downregulated in oocytes obtained from older women and the other nine miRNAs (let-7e-5p, miR-29a-3p, miR-126-3p, miR-136-5p, miR-192-5p, miR-203a-3p, miR-371-p, miR-484, and miR-494-3p) were upregulated [89]. The diminishment of ovarian reserve with age is associated with miRNAs and other non-coding RNAs, which may regulate age-associated changes in oocyte quality [90]. When miRNA expression profiling was performed using the follicular fluid of younger (<31 years) and older (>38 years) women, it was found that four miRNAs were significantly differentially expressed between the two groups. The predicted targets of these miRNAs were clearly enriched in genes involved in heparan-sulfate biosynthesis, extracellular matrix-receptor interactions, p53 signaling, and cytokine-cytokine-receptor interactions, with certain pathways already reported as determinants of reproductive aging in women [91]. Previous research has shown that miRNA expression profiles in the follicular fluid of patients with polycystic ovary syndrome or premature ovarian failure are different from those of normal individuals [92]. When miRNA and mRNA microarrays were used to measure biomarkers in the granulosa cells of women with POI, which is an extreme form of female reproductive aging, it was found that miR-379-5p was significantly upregulated. As miR-379-5p inhibits cell proliferation and attenuates the DNA repair function by directly targeting *PARP1* and *XRCC6*, these results corroborate the significance of DNA repair for POI and provide an epigenetic explanation for the disease [93]. Table 2 shows the miRNA in oocytes which undergo significant changes with an increase in age.

**Table 2:** Alterations in micro RNA expression of mechanism in the aged oocyte.

Gene name	Gene function	Ref.
miR-21-5p	human cumulus cell viability	[154]
miR-378	follicular development and oocyte maturation	[155]
miR-224	oocyte maturation	[156]
let-7	Ovarian arrest and blocked oocyte maturation	[157]
miR-130b	oocyte maturation and blastocyst formation	[158]
miR-451	Regulate embryo implantations sites	[159]
miR-143	Promotes FSH-induced estradiol production and granulosa cell proliferation	[160]
miR-574	Oocyte maturation	[161]
miR-375	Oocyte maturation	[162]
MiR-29	regulating differently various steroidogenic enzyme	[136]
miR-125a-3p	oocyte germinal vesicle breakdown	[163]

## 3. Effect of age on ovarian stroma

Aging is related to decreased efficiency of tissue remodeling and increased fibrosis, while ovulation and healing of ovarian wounds are impaired, with the development of advanced reproductive age. Moreover, tissue remodeling after ovulation will be damaged by age, such as decreased blood vessels in the corpus luteum, increased collagen, decreased hyaluronic acid, decreased cell proliferation and apoptosis, adipocyte hypertrophy, decreased number of adipocytes, and impaired wound healing. This is a new mechanism in addition to the decline in the number and quality of eggs, and may affect follicular development and oocyte quality [94-96]. The earliest stage of oocyte development not only plays a key role in age-related changes in gamete quality, but also has a large impact on the microenvironment of gamete development [97]. The results of single-cell RNA sequencing of oocytes from mice of different ages revealed the following in aged mice: changes in the protein metabolism of oocytes, significant changes in the expression of genes related to protein quality control (protein modifications and the unfolded protein response), cellular components related to protein metabolism are disrupted, differences in the expression of metabolism-related proteases, and significant increases in the expression of factors associated with inflammation and the number of ribosomes in the cytosol [98]. Consistent results with animals, ovarian tissue changes drastically during aging. Compared with young people, monocyte recruitment and macrophage replacement activation (M2) in the ovaries of the elderly are significantly increased. This suggests that the ovarian interstitial extracellular matrix is associated with fibrosis-related aging, and shows signs of chronic inflammation (inflammaging) related to aging, which may cause a significant decrease in the quality of oocytes in elderly individuals [99]. Therefore, there is close communication and integration between the follicle and its microenvironment, which may affect the quality of oocytes that grow during oogenesis.

## 4. Aging causes metabolic disorders in the ovaries

With increasing age, women may experience ovarian insufficiency, hypoestrogenemia, and a series of metabolic disorder-related symptoms, and the risk of developing metabolic syndrome becomes higher in women undergoing the menopausal transition. Previous research has revealed the occurrence of significant changes in the lipid content of the follicular fluid of aged women. Specifically, results have shown an increased abundance of sphingomyelin (whose metabolism constitutes a key event in apoptosis), diacylglycerol, and triacylglycerol (associated with reduced follicular maturation and oocyte quality) [100, 101]. Other studies have shown that the follicular fluid of aged women has lower glutathione peroxidase and superoxide dismutase levels [102, 103]. Advanced glycation end products (AGEs) are also closely related to declining ovarian function as the accumulation of AGEs contributes directly to protein damage, induces a chain of oxidative stress reactions, and increases inflammatory reactions, thereby inducing a premature decline in ovarian function [104, 105]. The external environment may affect aging by regulating key metabolic sensors (e.g., SIRT1 and AMPK), which interact with the mammalian target of rapamycin and insulin/insulin-like growth factor 1 (IGF-1) to control energy metabolism and cell growth. This reduces NAD^+^/NADH and AMP/ATP ratios, damages mitochondrial function, and increases oxidative stress [84, 106, 107]. Therefore, the quality of senescent oocytes treated with NAD^+^ metabolic precursor nicotinamide mononucleotide (NMN) was restored, and the quality of oocytes was improved by overexpression of the transgenic NAD^+^ dependent deacylase SIRT2. NMN supplementation can not only increase the ovulation of senescent oocytes, eliminate accumulated ROS and inhibit cell apoptosis, but also enhance their meiotic ability and fertilization ability by maintaining normal spindle structure and dynamics. Single-cell RNA sequencing of oocytes from C57BL/6 mice of different ages revealed the following in aged mice: changes in the protein metabolism of oocytes, significant changes in the expression of genes related to protein quality control (protein modifications and the unfolded protein response), disruption of cellular components related to protein metabolism, differences in the expression of metabolism-related proteases, and significant increases in the expression of factors associated with inflammation and the number of ribosomes in the cytosol [98]. A decline in ovarian function causes significant changes in the expression or modification of proteins such as maturation promoting factor, sirtuins (SIRT1/2/3), anti-apoptotic B-cell lymphoma 2 proteins, and caspase [108-110].

## 5. Telomere shortening causes oocyte aging

Telomeres are short tandem DNA repeats that bind members of the shelter protein complex to form a protective telomere ring, capping the ends of linear chromosomes. Insufficient number of telomere repeats can cause chromosomes to uncap, cell senescence, and death. Telomere length decreases with age, providing a surrogate indicator for biological age and predicting many age-related diseases [50, 111]. Telomere shortening serves as an important sign of human cell aging [112] and can be reversed through the expression of telomerase, an RNA-dependent DNA polymerase [50]. During the aging process of oocytes, telomere shortening also plays an essential role. A study by Turner and Hartshorne revealed that telomeres were significantly shorter in mature oocytes than those in immature oocytes. As unfertilized oocytes do not possess telomerase activity, the shorter telomere length in mature human oocytes may be attributed to progressive telomere shortening during the oogenesis process [113]. As repetitive DNA sequences with high GC content, telomeres are extremely sensitive to ROS. Even in undifferentiated cells such as oocytes, the presence of oxidative stress may result in telomere shortening [114]. Previous research has shown that extremely short telomeres may trigger a p53-dependent cell cycle arrest and cause DNA damage, which in turn activates cellular senescence pathways [115]. Another study indicated that the granulosa cells of women with primary ovarian insufficiency had lower telomerase activity and shorter telomeres, thereby demonstrating the potential application of telomerase activity and telomere length as molecular markers of ovarian insufficiency [116]. These results show that the normal aging process of oocytes is accompanied by telomere shortening, and pathological telomere shortening leads to the acceleration of oocyte aging. However, evidence showing that telomerase activation can reverse oocyte aging is lacking (Fig. 1).

## 6. Treatment strategies for ovarian insufficiency

### 6.4. Pharmacological alleviation

As mitochondrial dysfunction is related to ovarian insufficiency, the improvement of mitochondrial function may effectively alleviate or reverse ovarian insufficiency. Currently, mitochondrial supplements such as coenzyme Q10, resveratrol, rapamycin, α-lipoic acid, and SIRT3 are used in clinical practice to improve ovarian function [117]. Previous research has also indicated that omega-3 fatty acids can delay the decline in ovarian function and enhance oocyte quality. Certain pharmacological agents with oxidative stress-reducing, anti-inflammatory, and free radical-scavenging effects, such as c-phycocyanin or melatonin, may also potentially improve oocyte quality and enhance female fertility [118, 119].

In clinical practice, the antagonist protocol in patients with ovarian insufficiency leads to a similar number of retrieved oocytes compared with the long protocol. Although the short GnRH-a protocol is associated with a higher number of retrieved oocytes, its clinical outcome in pregnancy is not significantly different from that of other protocols [5]. Previous studies have reported that the oral administration of dehydroepiandrosterone starting from eight weeks before the ovulation induction cycle resulted in improved clinical outcomes in patients with ovarian insufficiency [120, 121]. Growth hormone supplementation during the antagonist intracytoplasmic sperm injection cycle can significantly increase the number of retrieved, fertilized, and transferable oocytes in women with inadequate ovarian response. However, it has no improvement effects on pregnancy and live birth rates [89]. Aging-induced ovarian insufficiency has a variety of causes and involves complex mechanisms. Despite the availability of many pharmacological agents that effectively alleviate the decline in ovarian function, drugs that can completely halt such a decline remain elusive.

### 6.5. Mitochondrial transplantation

Mitochondria play an important role in oocytes and may serve as the primary source of ATP during pre-implantation embryonic development. Mitochondrial dysfunction in oocytes has been regarded as a critical factor of poor oocyte development potential in older women. Various methods, including pharmacological treatment, cytoplasmic transplantation, nuclear transplantation, and mitochondrial transplantation, have been adopted to enhance mitochondrial integrity, activity, and quantity in aging oocytes. A study by Cohen et al. revealed that ooplasmic transfer in human oocytes could significantly improve embryonic development, promote pregnancy, and enable the birth of healthy children [122]. However, due to the complexity of the transplanted components and the existence of heteroplasmy, such an approach may lead to the creation of offspring with three genetic parents, which poses ethical issues. Therefore, the ooplasmic transfer procedure was suspended by the U.S. Food and Drug Administration in 2002 [123]. Mitochondrial transplantation involves the extraction of nuclear DNA from unfertilized oocytes of patients with mitochondrial abnormalities and subsequent transfer of the DNA to enucleated donor oocytes containing healthy mitochondria. The disadvantages of this method include poor clinical effectiveness and ethical issues arising from the co-existence of two different mtDNA genomes [124]. It has been reported that autologous mitochondrial transplantation can significantly improve oocyte quality and enhance the pregnancy success rate in older women [125]; however, other researchers have stated that autologous mitochondrial transplantation does not improve the quality of aged oocytes [126]. Therefore, the safety and effectiveness of mitochondrial transplantation require further validation.

## 7. Conclusion and outlook

Ovarian insufficiency is a key influencing factor of pregnancy. Aging affects the quality and function of various cells within ovaries, resulting in mitochondrial dysfunction, aneuploidy, metabolic disorders, and epigenetic modifications. Pharmacological agents that improve mitochondrial function, reduce oxidative stress, and enable free radical scavenging demonstrate limited therapeutic effectiveness and clinical interventional effects in treatment of ovarian insufficiency, while the effects and ethicality of mitochondrial transplantation remain debatable. Mesenchymal stem cells (MSCs) possess promising application aspects in clinical practice as preliminary experiments have indicated that MSCs can improve ovarian function and enhance the pregnancy rate in patients with ovarian insufficiency. However, large-scale clinical studies are required to validate the efficacy and safety of MSCs in patients with ovarian insufficiency. Delayed pregnancies and aging-induced ovarian insufficiency have affected the reproductive needs of an increasing number of women of childbearing age. At present, two major issues remain to be solved, namely the exact mechanisms by which aging causes a decline in oocyte quality and the development of feasible and effective treatment methods for attenuation of the aging-induced decline in ovarian function. With rapid advances in high-throughput technologies in fields such as epigenetics, transcriptomics, proteomics, and metabolomics, particularly the advancement of techniques for detection of multi-omics changes in single cells, our understanding of aging-induced ovarian insufficiency will be undoubtedly enhanced at an accelerated pace. Given the complex mechanisms of ovarian insufficiency and difficulties in clinical intervention, further research is needed to investigate ways to improve the ovarian function of patients with physiological and premature ovarian insufficiency and to elucidate the mechanisms by which aging affects the functions of cells within the ovaries.
